# Primary aortic intimal sarcoma masquerading as intramural hematoma

**DOI:** 10.1515/med-2021-0337

**Published:** 2021-09-03

**Authors:** Xiaodong Fan, Xiaofeng Chen, Zhiqi Yang, Tianhui Zhang, Yuting Liao, Weixiong Fan, Xiangguang Chen

**Affiliations:** Department of Radiology, Meizhou People’s Hospital, Meizhou, 514031, China; Guangdong Provincial Key Laboratory of Precision Medicine and Clinical Translational Research of Hakka Population, Meizhou, 514031, China; Guangdong Provincial Engineering and Technology Research Center for Molecular Diagnostics of Cardiovascular Diseases, Meizhou, 514031, China; GE Healthcare, Guangzhou, 510623, China

**Keywords:** thoracic aorta, intimal sarcoma, intramural hematoma

## Abstract

Primary aortic intimal sarcoma is a very rare disease and most patients present with features similar to those of atherosclerotic plaque and thrombus; however, primary aortic intimal sarcoma presents with features similar to those of intramural hematoma (IMH) on CT imaging and clinical presentation had never been previously reported. Here we report a case involving a 49-year-old woman with primary aortic intimal sarcoma masquerading as IMH on radiological images and clinical presentation. We also discuss some of the diagnostic pitfalls and hope that these diagnostic pitfalls will be very useful for clinicians.

## 1 Introduction

Primary aortic intimal sarcoma is a very rare disease with a poor prognosis [1,2]. The most frequently reported clinical symptoms are caused by embolic events, and most of them present with features similar to those of atherosclerotic plaque and thrombus [2,3,4,5,6,7]. Here we report a case of primary aortic intimal sarcoma masquerading as intramural hematoma (IMH) on CT imaging and clinical presentation and discuss the reasons for misdiagnosis.

## 2 Case presentation

**Figure 1:**
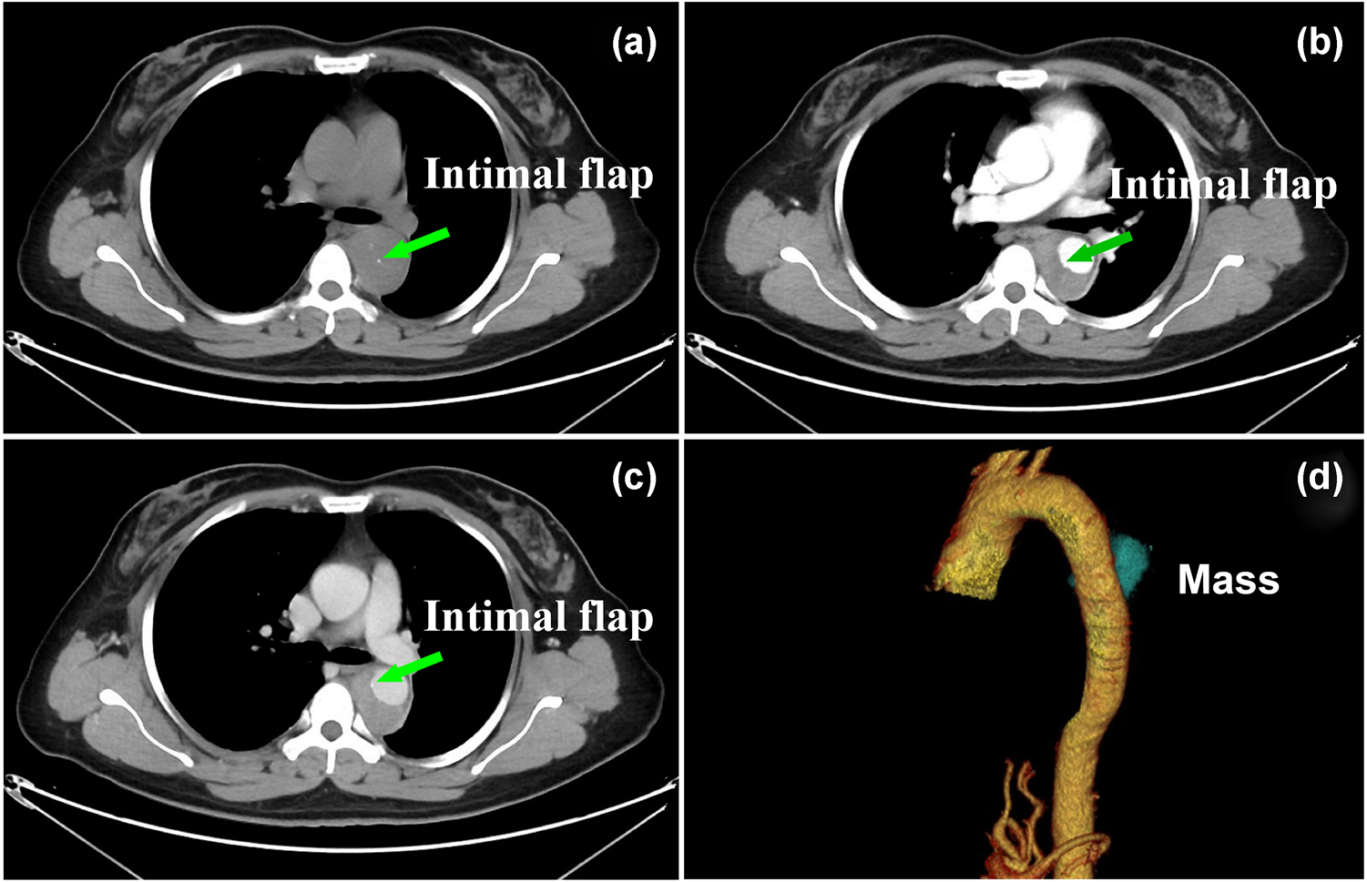
Axial nonenhanced CT images (a) showing a crescent-shaped thickening of aortic wall with the same attenuation as that of the lumen and intimal flap. CTA (c and d) showing an intimal flap and expanded false lumen with slight enhancement, and correspond reconstructed image (d) showing a mass-like false lumen, which was interpreted by radiologists as IMH.

**Figure 2:**
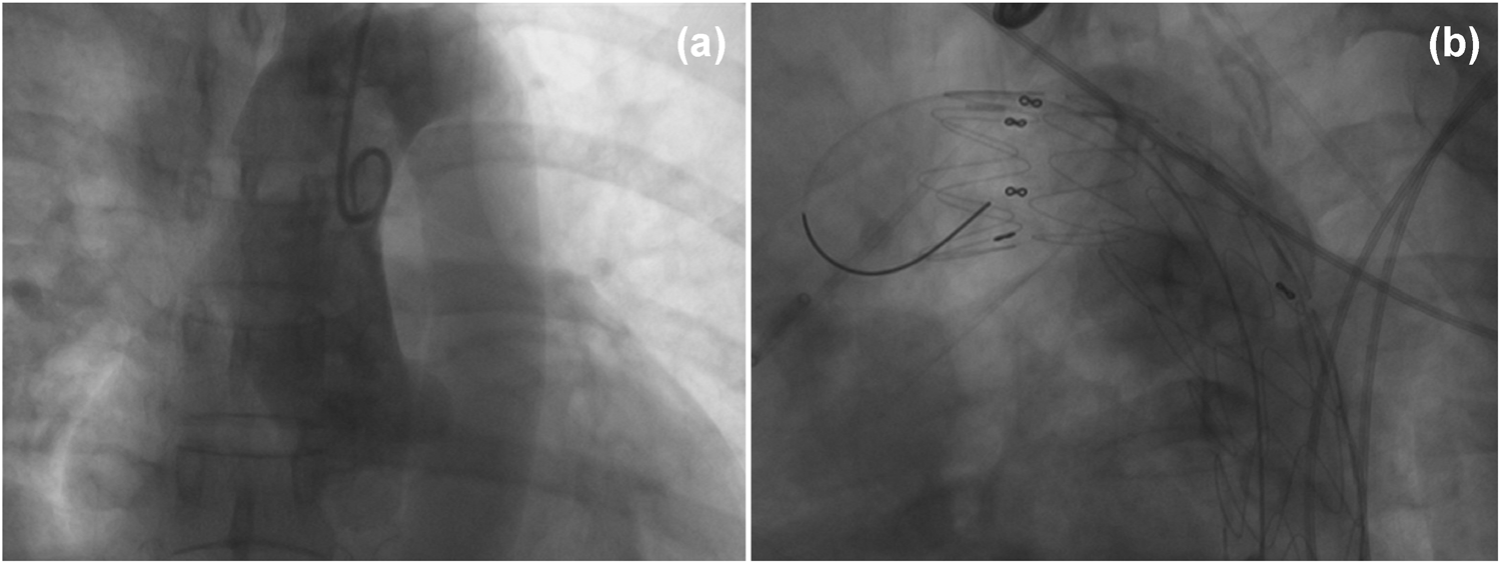
Digital subtraction angiography image showing mild stenosis in the thoracic aortic lesion without an obvious intimal flap (a) and the implanted stent (b).

A 49-year-old woman visited the emergency room for acute chest pain radiating to the back. She had known hypertension: the blood pressure in the left upper limb, right upper limb, left lower limb, and right lower limb was 210/65, 218/60, 176/90, and 175/90 mm Hg, respectively. The D-dimer level was normal at initial admission. An electrocardiogram showed a normal sinus rhythm. Considering that the patient had typical clinical symptoms of acute chest pain radiating to the back, with asymmetry of limb blood pressures, and a normal D-dimer level and electrocardiogram, the diagnosis of IMH was suspected. Chest nonenhanced computed tomography (CT) was performed, which showed crescent-shaped thickening of the thoracic aorta wall with the same attenuation as that of the lumen and linear calcification ingression (Figure 1a). Computed tomography angiography (CTA) revealed an intimal flap and expanded false lumen with slight enhancement (Figure 1b–d). Digital subtraction angiography showed mild stenosis in the thoracic aortic lesion without an obvious intimal flap (Figure 2a). Based on both the clinical symptoms and diagnostic test results, only thoracic IMH was considered by both radiologists and cardiothoracic surgeons; hence, thoracic endovascular aortic repair and not surgery was considered at that time. Then, an endovascular stent (30 mm × 200 mm, Medtronic) was implanted into the patient. Completion angiography demonstrated that the thoracic aorta and branches were patent, with no obvious endoleak or extravasation (Figure 2b). The prevalence of acute chest pain radiating to the back decreased after treatment, and the patient recovered well and was discharged home.

**Figure 3:**
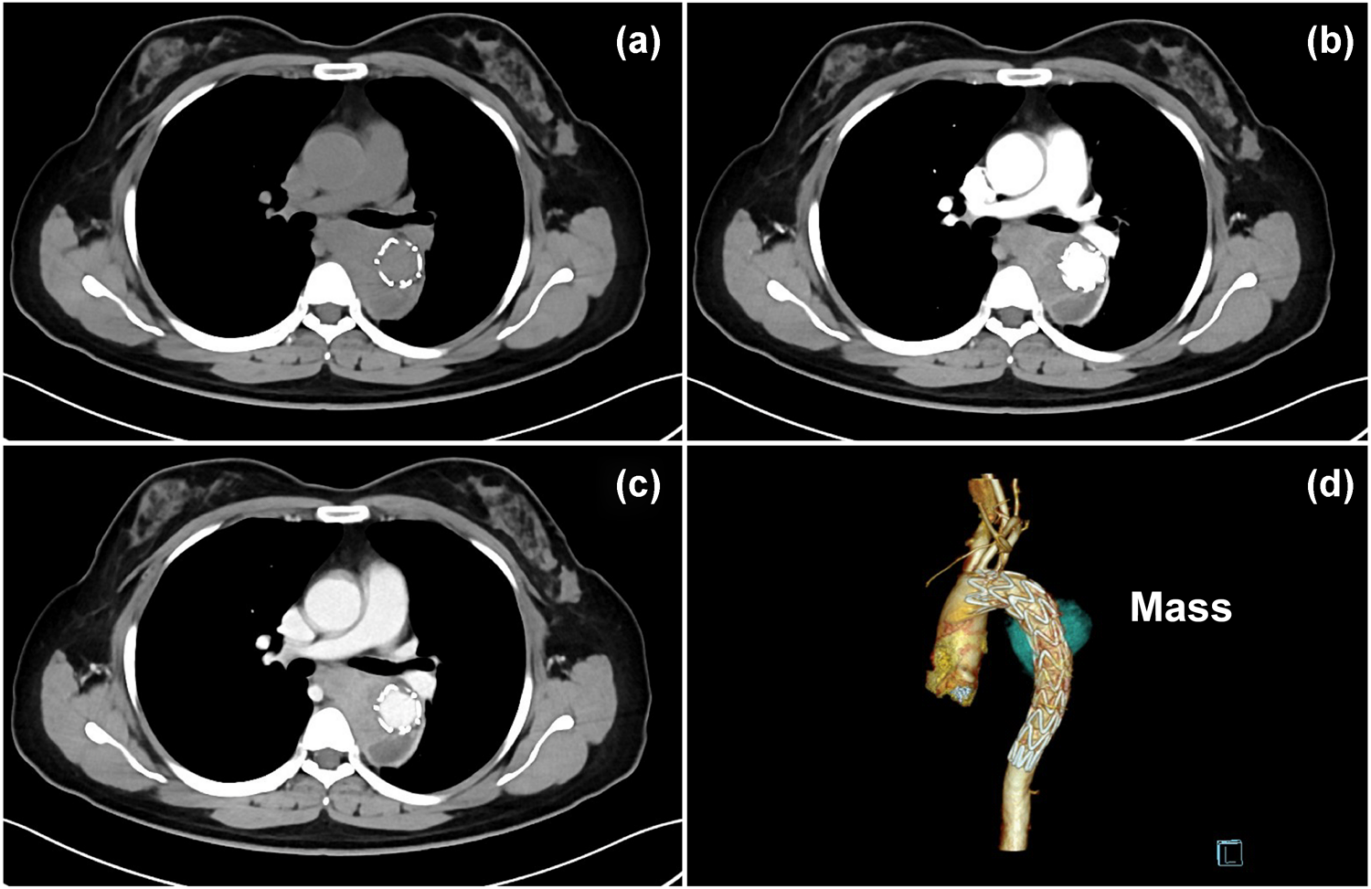
Axial nonenhanced CT image (a) and CTA (b–d) showing enlargement of the false lumen three months after stent implantation, with nonhomogeneous enhancement and mediastinal extensions of the false lumen, which was interpreted by radiologists in our hospital and other medical centers during follow-up as an endoleak and periaortic hematoma.

**Figure 4:**
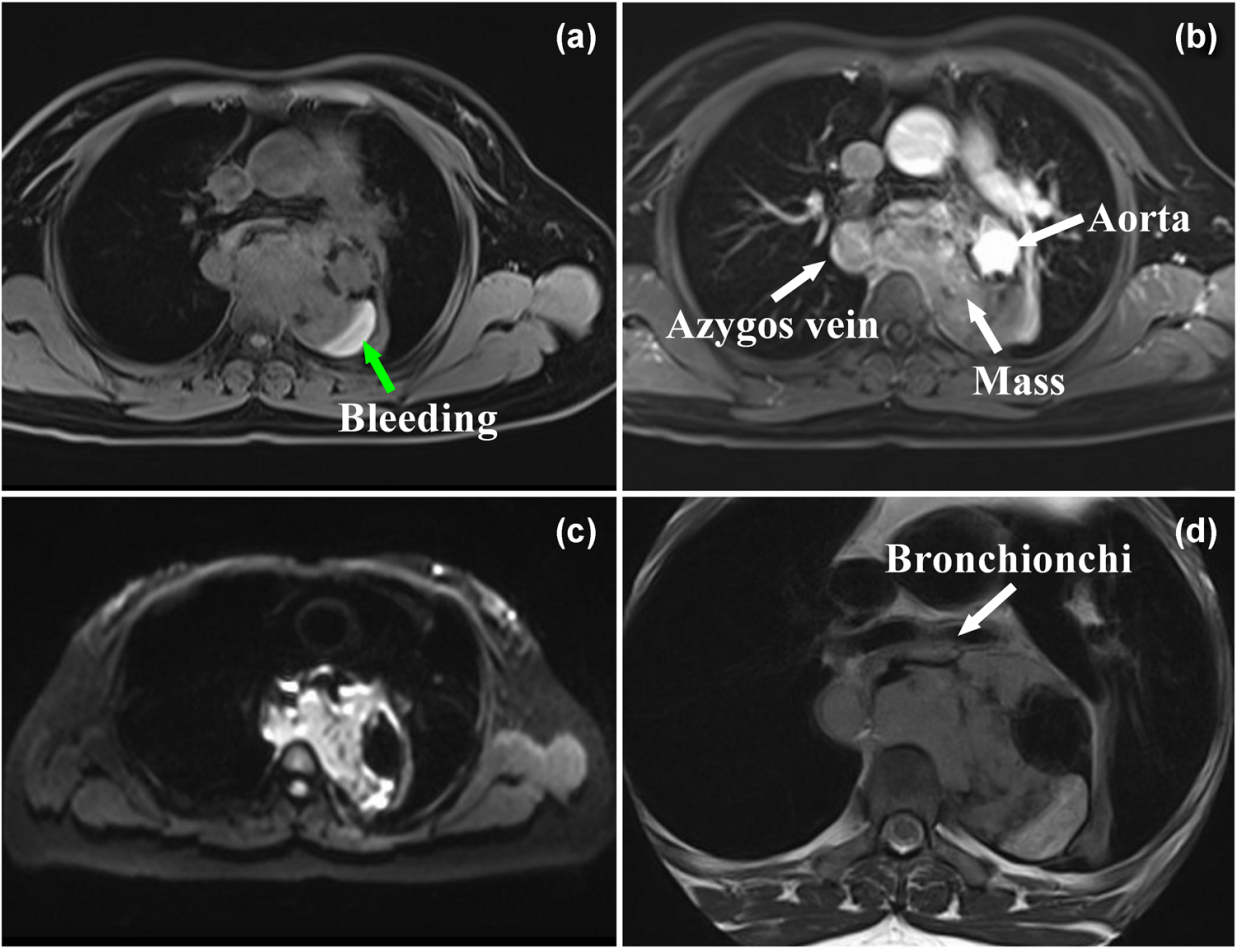
Magnetic resonance imaging showing gradual enlargement of false lumen with mediastinal extensions and a new lesion in the azygos vein. T_1_WI image (a) and T_2_WI image (d) showing the heterogeneous of “false lumen” with bleeding area (green arrow). Contrast-enhanced T_1_WI image (b) showing a nonhomogeneous enhancement of the “false lumen,” which correspond to the hyperintense regions in DWI (*b* = 800 s/mm^2^) image (c).

**Figure 5:**
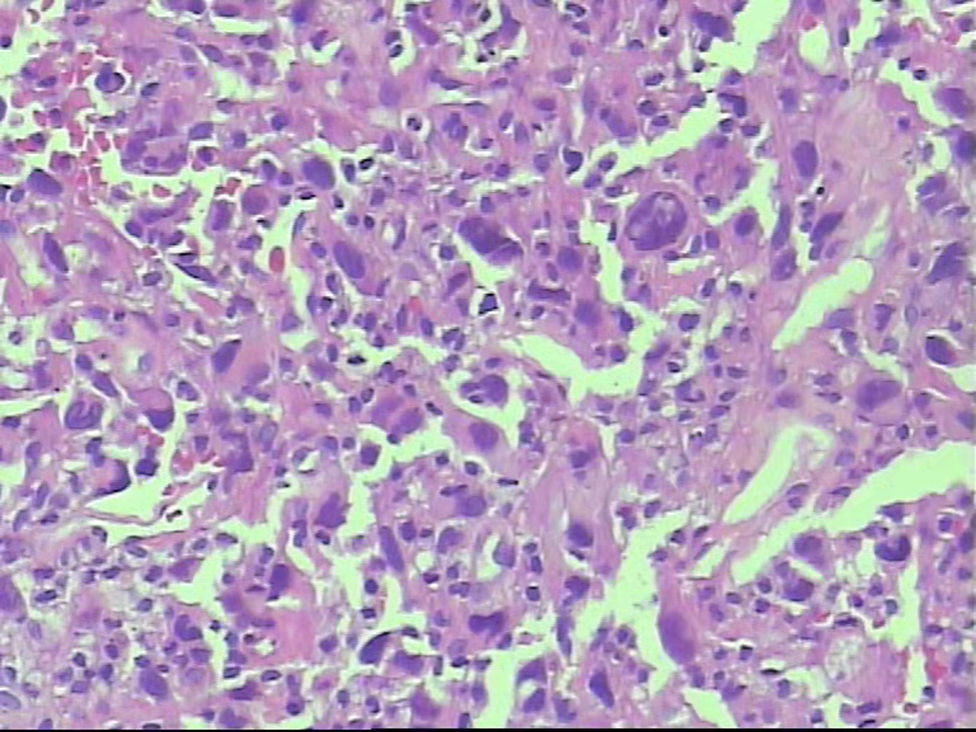
Histopathology of aortic tissues showed that the tumor was composed of malignant spindle cells and demonstrated nuclear pleomorphism and atypia (hematoxylin and eosin staining ×200).

Three months after endovascular stent graft implantation, follow-up CTA (Figure 3) showed enlargement of the false lumen with nonhomogeneous enhancement and mediastinal extensions. The patient was interpreted by radiologists as having an endoleak and periaortic/mediastinal hematoma, which were also considered by other medical centers during follow-up. However, seven months later, the patient was readmitted to the hospital with complaints of dysphagia and dyspnea. Subsequent magnetic resonance imaging (Figure 4) showed gradual enlargement of the false lumen with mediastinal extensions and new lesions in the azygos vein. Angiogenic hemangioma was suspected, and endobronchial ultrasound biopsy was performed to confirm the lesion. Histopathological evaluation indicated an intimal sarcoma (Figure 5). Immunohistochemical staining for vimentin, CD10, CD68, and CD99 was positive, whereas that for S-100, CD56, desmin, CD30, CD15, leukocyte common antigen, smooth muscle actin, CD34, CD61, and CD5 was negative. Afterwards, she received adjuvant chemotherapy with a combined treatment of ifosfamide and epirubicin; however, the general condition of the patient rapidly deteriorated after 1 cycle of adjuvant chemoradiotherapy, and she died exactly 19 months after the initial hospitalization.

**Informed consent:** Informed consent was obtained from the patient’s family.

## 3 Discussion

Primary aortic intimal sarcoma can be diagnosed only by postoperative histopathology and immunohistochemical markers, while its preoperative diagnosis is challenging because of various clinical manifestations and no specific imaging features. Based on previous case reports [2,3,4,5,6,7], aortic intimal sarcoma often presents with features similar to those of atherosclerotic plaque and thrombus on CT images, and the most frequently reported clinical symptoms are caused by embolic events. In contrast to prior studies, acute chest and back pain with asymmetry of limb blood pressures and an expanded false lumen with an intimal flap were the only features observed on CT images. Thus, as these features were similar to those of IMH, this led to the confident diagnosis of “mimicking IMH.” An episode of acute chest and back pains might be explained by micro-calcification/micro-hemorrhage of the tumor, which showed as a hyperintense foci at the center of the aorta on the non-contrast CT (Figure 1a and b) and that was interpreted by radiologists as “intimal flap.”

According to the previously reported cases, the diagnosis of IMH depends on the identification of the intimal tear, the false lumen, and the presence of most hyperdense intramural thrombus on unenhanced images [8,9,10,11]. However, their following features differed from those on previous reports of IMH: First, the “false lumen” was limited in length and made it seem more like a mass, although it had an intimal flap on CT images. Second, in contrast to the typical feature of obviously enhanced blood in the false lumen, the false lumen was filled with a slight and nonhomogeneous enhancement mass. Third, the “false lumen” tended to grow extravascularly, which manifests as an aggressive tumor. In this case, stent implantation might have further facilitated sarcoma invasion into the mediastinum. Fourth, the aortogram demonstrated mild stenosis in the thoracic aorta lesion without severe atherosclerotic changes in the aortic wall and obvious intimal flap. Furthermore, the following features with regard to a leak and periaortic hematoma after stent graft implantation differed from those on previous reports [12,13]: first, there was no open channel inside the stent graft and no obvious backflow in the “false lumen.” Second, the “false lumen” gradually became enlarged, increasing the tendency of mediastinal invasion. Third, MRI showed a heterogeneous mass with a false lumen and a new lesion in the azygos vein. To our knowledge, no similar case has previously been reported in the English and Chinese literature.

According to previous studies, the prognosis of primary aortic intimal sarcoma is dismal [1,14,15,16,17,18]. Consistent with these studies, the overall survival in this case was 19 months, even after treatment with adjuvant chemotherapy. Therefore, clinicians must be vigilant for primary aortic intimal sarcoma in patients with IMH manifesting as acute chest and back pains, a localized false lumen and intimal flap, slight and nonhomogeneous enhancement of the false lumen, and extravascular extensions on CT images.
